# Health Promotion Programs and Policies in the Workplace: An Exploratory Study With Alaska Businesses

**DOI:** 10.5888/pcd17.200111

**Published:** 2020-10-15

**Authors:** Craig N. Sawchuk, Joan Russo, Gary Ferguson, Jennifer Williamson, Janice A. Sabin, Jack Goldberg, Odile Madesclaire, Olivia E. Bogucki, Dedra Buchwald

**Affiliations:** 1 Department of Psychiatry and Psychology, Mayo Clinic, Rochester, Minnesota; 2 Department of Psychiatry and Behavioral Sciences, University of Washington, Seattle, Washington; 3 University of Alaska, Anchorage, Alaska; 4 Alaska Native Tribal Health Consortium, Anchorage, Alaska; 5 Department of Biomedical Informatics and Medical Education, University of Washington, Seattle, Washington; 6 Department of Epidemiology, University of Washington, Seattle, Washington; 7 Institute for Research and Education, Washington State University, Spokane, Washington

## Abstract

**Introduction:**

We examined health insurance benefits, workplace policies, and health promotion programs in small to midsize businesses in Alaska whose workforces were at least 20% Alaska Native. Participating businesses were enrolled in a randomized trial to improve health promotion efforts.

**Methods:**

Twenty-six Alaska businesses completed from January 2009 through October 2010 a 30-item survey on health benefits, policies, and programs in the workplace. We generated frequency statistics to describe overall insurance coverage, and to detail insurance coverage, company policies, and workplace programs in 3 domains: tobacco use, physical activity and nutrition, and disease screening and management.

**Results:**

Businesses varied in the number of employees (mean, 250; median, 121; range, 41–1,200). Most businesses offered at least partial health insurance for full-time employees and their dependents. Businesses completely banned tobacco in the workplace, and insurance coverage for tobacco cessation was limited. Eighteen had onsite food vendors, yet fewer than 6 businesses offered healthy food options, and even fewer offered them at competitive prices. Cancer screening and treatment were the health benefits most commonly covered by insurance.

**Conclusion:**

Although insurance coverage and workplace policies for chronic disease screening and management were widely available, significant opportunities remain for Alaska businesses to collaborate with federal, state, and community organizations on health promotion efforts to reduce the risk of chronic illness among their employees.

**Summary**

**What is already known on this topic?**

Workplace wellness programs have demonstrated effectiveness on policy changes and employee health outcomes.

**What is added by this report?**

We describe an adaptation of the American Cancer Society’s Workplace Solutions Program to small- to midsize Alaskan businesses whose workforce is made up of approximately 20% Alaska Natives.

**What are the implications for public health practice?**

Collaborating with the business sector will be essential in meeting the goals of Healthy People 2020.

## Introduction

Cancer and cardiovascular disease are the leading causes of death in Alaska (1), as in the rest of the United States (2). Compared with the general population, however, Alaska residents have higher rates of obesity, cigarette smoking, and excess alcohol use and limited engagement in physical activity and consumption of fruits and vegetables (3,4), all of which are risk factors for chronic disease (5). Further disparities have been identified among the various racial and ethnic groups in Alaska. Relative to non-Hispanic White residents, for example, Alaska Natives have higher mortality rates, higher rates of and risk factors for chronic disease, and less access to health care (6,7).

Recognizing the need for chronic disease management at the population level, the US government set national goals for improving health and preventing disease. These are summarized in the guidelines for Healthy People 2020 (8) and will be expanded in the forthcoming Healthy People 2030 (9). Achieving these goals requires collaboration with federal, state, and community-based organizations that have the capacity to develop health promotion programs (10,11). The American Cancer Society, the American Diabetes Association, and the Substance Abuse and Mental Health Services Administration are examples of national organizations developing and disseminating evidence-based health interventions. The Alaska Department of Health and Social Services Division of Public Health similarly organizes a variety of state and local resources, such as the Alaska Diabetes Prevention and Control Program and the Alaska Tobacco Prevention and Control Program, which can assist in achieving the goals of Healthy People 2020 and will extend to Healthy People 2030.

The workplace is an important venue for establishing and delivering collaborative health programs. Businesses can institute organizational policies, develop workplace programs, and shape employee insurance coverage to encourage better health (12,13). Investing resources in evidence-based health promotion can be advantageous to employers by lowering health care costs, reducing sick days, and improving workforce productivity. Given these advantages, the American Cancer Society developed an evidence-based package of strategies for health promotion and chronic disease management known as Workplace Solutions, which was designed for implementation in employment settings across the United States.

The Workplace Solutions package identifies best practices in several areas, including health insurance benefits (eg, providing full coverage for cancer screening), workplace policies (eg, banning tobacco in the workplace), workplace programs (eg, offering physical activity programs in the workplace), employee tracking (eg, surveying employees’ health behaviors to track the effectiveness of health promotion efforts), and company-wide communication (eg, conducting targeted health promotion campaigns) (14). Initial studies of Workplace Solutions found that these efforts were acceptable to employers and led to the adoption of programs to manage chronic disease (14,15).

The objective of our study was to describe health promotion programs and policies in place at several small to midsize Alaska businesses (N = 26) that enrolled in a randomized trial to assess the feasibility of implementing Alaska Workplace Solutions, an adaptation of Workplace Solutions. We conducted this descriptive study with Alaska businesses to understand possible innovation points of intervention for improving the health of the Alaska small business workforce and to help inform feasible intervention efforts.

## Methods

### Study population and procedures

Our study was conducted in the context of a large intervention trial of Alaska Workplace Solutions. For the parent study, we identified an initial pool of 385 small (<250 employees) to midsize (>250 employees) workplaces across Alaska. At recruitment, small to midsize workplaces were defined as employing a workforce of 50 to 1,000. This definition was expanded midway through the study to 40 to 1,500 employees to increase the number of eligible businesses. To be eligible for the parent intervention trial, businesses had to employ workforces that were at least 20% Alaska Native, have been in existence for at least 3 years, and be willing to be participate in either an immediate or a delayed intervention. Businesses were recruited by using a 3-step process, which included mailing businesses informational material, directly contacting businesses to answer questions and assess interest in and eligibility for the study, and meeting with businesses in person to present information about the study and answer questions before enrollment. Most businesses either failed to meet these eligibility criteria, declined study participation, or discontinued communication with our study team. The final Alaska Workplace Solutions sample consisted of 26 businesses.

The Alaska Native Tribal Health Consortium and the University of Washington collaborated on all aspects of design, recruitment, and implementation for the parent intervention trial. We adapted the evidence-based health promotion and chronic disease prevention strategies developed by the American Cancer Society for Alaska-based workplaces. A full report of that trial is in preparation. Our study consisted solely of our initial workplace survey. After reviewing a memorandum of understanding with each participating business, we administered a baseline questionnaire to each participant to assess company and workforce characteristics and current health-related programs and policies. Surveys were administered from January 2009 through October 2010. A company representative (eg, chief executive officer, human resource manager, supervisor) who served as the study liaison completed the baseline survey and returned it by fax or email. As part of the parent study, businesses that completed the baseline questionnaire were then randomized to the intervention or control group.

### Measures

A 30-item survey adapted from the American Cancer Society Workplace Program (14) assessed basic characteristics of the workplace, insurance coverage, company policies, and workplace programs for employee health promotion and disease management. Companies had the option of completing the survey by telephone, fax, e-mail, or secure website.

### Statistical analyses

We calculated descriptive statistics on workforce demographics and generated frequency statistics to describe overall insurance coverage and availability and existing coverage, policies, and programs for the following health-related domains 1): tobacco 2); physical activity and nutrition, and 3) disease screening and management.

## Results

**Workplace characteristics**. Most of the 26 Alaska-based businesses that participated in the study operated in the sectors of company management (n = 7), government (n = 6), and health care/social assistance (n = 6). The average number of contacts, which included email, telephone calls, and in-person visits, required to enroll workplaces was 25 (range,10–30). Companies employed an average of 250 workers (SD, 294; median, 121), although workforces ranged in size (range, 41–1,200). Most employees worked full time (mean, 84%; SD, 11%) and were Alaska Natives (mean, 57%; SD, 23%). About half of the sample (mean, 48%; SD, 23%) had some level of post-high school education. Twenty of 26 companies provided employee salary data. The average employee annual salary among these businesses was approximately $55,000.

**Baseline coverage, policies, and programs**. We saw several similarities in health insurance coverage across employers (Table 1). Most companies offered insurance for employees and their families, and all paid at least 50% of insurance costs for full-time employees. Almost all provided some form of prescription drug coverage for full-time employees and their families. Twenty-one of 26 companies offered at least partial coverage for the cost of transportation to obtain health care. However, few companies sent health reminders to employees, informed network providers of employees’ use of preventive care, or tracked delivery of preventive care.

**Table 1:** Health Insurance Coverage Among Alaska-Based Businesses (N = 26), January 2009 to October 2010

Insurance Coverage	n
Company pays at least 50% of total insurance cost for full-time employees	26
Company pays at least 50% of total insurance cost for families of full-time employees	23
Company provides prescription drug coverage for employees	24
Company provides prescription drug coverage for families of employees	25
Company covers transportation costs for health care	21
Company sends employees age-appropriate health care reminders	3
Company sends preventive care service reminders to network health care providers	1
Company tracks delivery of preventive care services	2

Insurance coverage, policies, and programs for tobacco, physical activity and nutrition, and chronic disease screening and management differed by employer (Table 2). Regarding tobacco cessation, few companies offered full insurance coverage for prescription medication (n = 4), nonprescription nicotine replacement therapy (n = 4), or counseling for tobacco cessation (n = 4). Slightly more coverage was available for nicotine replacement therapy (n = 5) and counseling (n = 8) if employees shared the expense through out-of-pocket payments, and 15 companies offered prescription medication for smoking cessation with employee co-payment. Twelve companies offered referrals for tobacco cessation assistance, with 11 offering telephone-based therapy or a quit line. A total of 19 businesses had a policy restricting tobacco use, but only 6 completely banned tobacco use on company premises.

**Table 2:** Health Insurance Coverage, Policies, and Programs for Tobacco; Physical Activity and Nutrition; and Disease Screening and Management at Alaska-Based Businesses (N = 26), January 2009-October 2010

Variable	n
**Tobacco**	
Insurance coverage for prescription medication for smoking cessation with no out-of-pocket cost	4
Insurance coverage for prescription medication for smoking cessation with out-of-pocket cost	15
Insurance coverage for over-the-counter nicotine replacement therapy with no out-of-pocket cost	4
Insurance coverage for over-the-counter nicotine replacement therapy with out-of-pocket cost	5
Insurance coverage for face-to-face tobacco cessation counseling with no out-of-pocket cost	4
Insurance coverage for face-to-face tobacco cessation counseling with out-of-pocket cost	8
Referrals for tobacco cessation assistance	12
Access to telephone-based tobacco cessation program or quit line	11
Onsite group program for tobacco cessation	1
Company policies restricting tobacco use at the workplace	19
Company policies banning tobacco use at the workplace	6
**Physical activity and nutrition**	
Company policy or resources for physical activity	5
Access to physical activity facilities at workplace or nearby	13
Policy allowing employees to exercise during work hours	2
Discounts or financial incentives to join commercial fitness centers	7
Company-organized or sponsored physical activity program within the past year	11
Company policy for food and nutrition standards	1
Food available onsite (cafeterias, vending machines)	18
Healthy food choices (eg, fruits, vegetables, low calorie) available onsite	6
Healthy food choices subsidized or competitively priced	3
Company-organized or sponsored weight control or healthy eating program	8
**Disease screening and management**	
Insurance coverage for cancer treatment	25
Insurance coverage for cancer clinical trial participation	9
Insurance coverage for breast cancer screening with no out-of-pocket cost	18
Insurance coverage for colon cancer screening with no out-of-pocket cost	10
Insurance coverage for colon cancer screening with out-of-pocket cost	15
Insurance coverage for cervical cancer screening with no out-of-pocket cost	17
Insurance coverage for influenza vaccination with no out-of-pocket cost	14
Insurance coverage for influenza vaccination with out-of-pocket cost	7
Onsite influenza vaccinations	13
Policy requiring sun protection for outdoor workers	0
Sunscreen or protective clothing provided for outdoor workers	2

Although 13 companies offered some type of facility or resource for employees’ physical activity, few had formal policies on physical activity, and only 2 allowed employees to take time off to exercise during the work day. Incentives and discounts to join local exercise facilities were offered by 7 companies. Eleven businesses sponsored a physical activity program during the previous year.

Although most companies offered food onsite, 6 offered healthy food choices, and only 3 had healthy foods subsidized or priced to make them more affordable. Few companies had formal policies governing workplace nutritional standards, and 8 sponsored some type of weight control or healthy eating program in the workplace.

Twenty-five companies offered insurance coverage for cancer treatment. Nine businesses provided some form of coverage to participate in clinical trials for cancer management. Full coverage for screening was most frequent for breast cancer. Coverage for colon cancer screening, even with out-of-pocket employee contributions, was less frequent than coverage for breast cancer screening. Full coverage for cervical cancer screening was available in 17 companies. Thirteen companies provided onsite influenza vaccinations, and 21 provided either full or partial coverage for the costs of offsite influenza vaccination. No business in our survey had policies requiring employees to use sun protection when working outdoors, and only 2 provided sunscreen or protective clothing for outdoor workers. However, these numbers should be interpreted in light of Alaska’s cool climate and geographic location in high latitudes where sun exposure is not a substantial issue. Also, our survey did not ask businesses whether any of their employees worked outdoors.

## Discussion

Workplaces are excellent settings for chronic disease management, given the ability of companies to directly communicate with employees, set policies on employee behavior, and provide insurance coverage (12). Programs and policies that promote disease management by reducing risk factors, introducing wellness initiatives, and providing effective health coverage are advantageous to employers and employees alike. Our survey of small to midsize Alaska businesses found that some form of individual and family insurance coverage was available for most employees, especially full-time workers. Reviews of the literature have indicated that people with health insurance use preventive services more often, are more likely to obtain treatment for chronic health conditions, and have better health outcomes than those without (16,17). Insurance can reduce or eliminate out-of-pocket medical costs, thereby addressing financial barriers to preventive and long-term care.

The relatively high number of Alaska businesses offering coverage for health care–related transportation costs was encouraging, given Alaska’s vast area and extreme weather, which can present major barriers to health care access. However, we administered only a global measure of the availability of transportation benefits, so it is unclear whether both full and partial travel coverage equally predict health care use.

As in previous workplace studies (14), few businesses in our Alaska sample had systems to send preventive service reminders to employees or track delivery of preventive care. Such systems may be challenging to implement, because they often require collaboration and negotiation with insurance carriers to find practical ways to share data on employee’s health care use. However, technologies such as electronic medical records and health registry databases can assist in creating standardized systems to generate automated health service reminders and track use of preventive services. Although upgrades in technological infrastructure are costly, they may pay for themselves over time if they help to maintain a healthier workforce with fewer sick days and improved worker productivity.

Rates of tobacco use are particularly high among Alaska Natives and Non-Hispanic White Alaskans (18). Most workplaces participating in our study had policies restricting tobacco use, but only 6 implemented a full tobacco ban. In addition, the extent and type of tobacco cessation programs varied considerably. The proportion of companies offering full insurance coverage for cessation options was small; more frequent was partial coverage with out-of-pocket payments by employees. It is possible that variability among the types of industries in our sample could have influenced patterns of insurance coverage and benefits available. Tobacco control programs, for example, may be particularly relevant for a business sector in which the baseline rates of tobacco use among employees are higher than that of the average population.

A recent review of workplace smoking cessation programs found that group therapy, individual counseling, pharmacotherapy, and multiple intervention programs were all associated with higher cessation rates than minimal treatment or none at all (19). Another study found that interventions combining behavioral treatment and pharmacotherapy tend to be more effective than either usual care or low-intensity behavioral treatment (20). However, small companies and companies in remote locations may have difficulty providing combined cessation strategies. They may need to rely on low-intensity or virtual quit options, such as medication management and free state-run telephone quit lines. Networking with the Alaska Tobacco Prevention and Control Program through the Alaska Division of Public Health may be one such avenue to support business in offering tangible, low-cost, smoking cessation resources. Previous workplace studies have found that tobacco cessation programs are among the most commonly adopted chronic disease prevention interventions (14). Given the well-documented hazards of tobacco use, improving tobacco cessation coverage in the workforce may be the best single strategy to improve employee health.

Many businesses had policies and resources relevant to physical activity and nutrition, providing a good foundation for future health promotion campaigns. Thirteen businesses in our study had physical activity facilities either onsite or nearby, and 11 had recently sponsored a physical activity program for employees. However, few offered healthy food choices, and fewer still at competitive prices. Subsidizing healthy food may be cost-prohibitive for small and geographically remote businesses. Cost-control strategies, such as networking businesses to negotiate lower food prices with vendors, may be necessary.

Rates of obesity in Alaska are high (21), and the annual economic impact of obesity on employers is profound (22). Alaska businesses are likely to benefit from evidence-based weight-loss programs that are low-cost, employee-managed, and clinically effective. One such approach is the Diabetes Prevention Program (23), which was developed with support from the National Institutes of Health as a lifestyle intervention targeting weight loss through physical activity, changes in nutrition, and self-management. It has been adapted for employment settings (FUEL Your Life), with a simplified, largely self-directed emphasis that led to moderate but significant weight loss in the pilot program and weight maintenance in a subsequent study (24,25).

Among all businesses, disease screening and management were the domains most frequently addressed by insurance coverage, policies, and programs, especially for cancer treatment. Although workplace wellness interventions still have room to improve, it is encouraging that of the 26 businesses participating, most fully covered screening for breast cancer (n = 17) and cervical cancer (n = 18), 10 businesses fully covered colon screening, and 13 worksites offered influenza vaccinations. Businesses that covered one type of cancer screening and management likely also covered other types of cancer.

Our study has several limitations. First, the number of eligible businesses in our sample was small. This may limit the generalizability of our results to other Alaska-based employers and hinder more detailed comparisons between businesses of different sizes and varying percentages of Alaska Native employees. A more detailed analysis might determine whether the types of health promotion programs and insurance coverage available differed by business size and geographic location. Second, our sample may be subject to selection bias, insofar as businesses with more stable infrastructure may have been more capable of providing the information we requested on insurance coverage and policies. Third, we had no access to employee-specific data, such as the individual frequency of smoking or use of available benefits, and thus objective validation of questionnaire responses was not possible. Nevertheless, a focus on employer practices rather than employee behaviors is a common limitation in research on worksite health promotion (14,24). However, a larger-scale, randomized workplace wellness trial failed to note any significant improvements in objective measures of employee health, work productivity, and health care use (13). Fourth, we did not assess any factors (eg, cost, logistics) that might explain why companies did not offer certain chronic disease management programs. Likewise, the baseline questionnaire only assessed insurance coverage for full-time employees, and therefore, the insurance status of part-time employees was not available for a more refined analysis. Fifth, our study was based on the American Cancer Society Workplace Solutions program, which emphasized risk factors and resources related to cancer. We did not assess other notable risk factors, such as alcohol consumption, and chronic diseases, such as cardiovascular disease, cerebrovascular disease, or diabetes, which are relevant to the Alaska population. Finally, our study was conducted from January 2009 through October 2010. The Affordable Care Act, which was passed in March 2010, increased the accessibility and affordability of preventive services (26). Although many employees in our sample were insured through a company plan, the Affordable Care Act likely expanded coverage among uninsured employees and made these wellness, health care, and preventive services more affordable for employees. This may have increased the accessibility of certain services (eg, tobacco cessation, breast and colon cancer screening, influenza vaccination) after completion of our survey.

To our knowledge, ours is the first study of its kind to assess workplace programs and policies in Alaska-based businesses that employ Alaska Natives. Supported by the American Cancer Society, this study adds to our understanding of environments where interventions can promote wellness and reduce chronic disease risk in a historically understudied population. Likewise, recruiting and retaining smaller and minority-owned businesses can be a challenging, yet important, venture for future health-promotion efforts in workplace wellness interventions (27,28). The National Institute for Occupational Safety and Health\Total Worker Health model may be a particularly important framework to use in future studies addressing workplace health promotion efforts. Launched in 2011, Total Worker Health brings together policies, programs, and practices that integrate protection from work-related safety and health hazards with promotion of injury and illness prevention efforts to advance worker well-being (29). This model highlights the importance of environmental influences on health and elucidates how evidence-based interventions can be implemented to support and promote better employee health.

A primary goal of Healthy People 2020 and likely Healthy People 2030 is to develop population-based approaches to disease management. Expanding knowledge of and collaboration with federal, state, and local health promotion resources with worksites is an excellent strategy to reach this goal. Initial studies of Workplace Solutions yielded results that can help businesses play a bigger role in managing chronic disease (14,15). The geographical, financial, and cultural landscape of Alaska is very different from the rest of the United States, presenting unique opportunities for creative adaptations of workplace health programs.
